# Water use efficiency across scales: from genes to landscapes

**DOI:** 10.1093/jxb/erad052

**Published:** 2023-02-13

**Authors:** Vincent Vadez, Raphael Pilloni, Alexandre Grondin, Amir Hajjarpoor, Hatem Belhouchette, Youssef Brouziyne, Ghani Chehbouni, Mohamed Hakim Kharrou, Rim Zitouna-Chebbi, Insaf Mekki, Jérôme Molénat, Frédéric Jacob, Jérôme Bossuet

**Affiliations:** French National Research Institute for Sustainable Development (IRD), UMR DIADE, University of Montpellier, 911 Av. Agropolis BP65401, 34394, Montpellier, France; International Crops Research Institute for the Semi-Arid Tropics (ICRISAT), Patancheru, 502 324, Telangana, India; LMI LAPSE, CERAAS-ISRA, Thiès, Senegal; French National Research Institute for Sustainable Development (IRD), UMR DIADE, University of Montpellier, 911 Av. Agropolis BP65401, 34394, Montpellier, France; French National Research Institute for Sustainable Development (IRD), UMR DIADE, University of Montpellier, 911 Av. Agropolis BP65401, 34394, Montpellier, France; French National Research Institute for Sustainable Development (IRD), UMR DIADE, University of Montpellier, 911 Av. Agropolis BP65401, 34394, Montpellier, France; ABSys, Université de Montpellier, CIHEAM-IAMM, CIRAD, INRAE, Institut Agro, Montpellier, France; International Water Management Institute (IWMI), MENA Office, Giza 12661, Egypt; International Water Research Institute (IWRI), Mohammed VI Polytechnic University (UM6P) UMR CESBIO, Benguerir 43150, Morocco; International Water Research Institute (IWRI), Mohammed VI Polytechnic University (UM6P) UMR CESBIO, Benguerir 43150, Morocco; INRGREF, Carthage University, B.P. 10, 2080 Ariana, Tunisia; INRGREF, Carthage University, B.P. 10, 2080 Ariana, Tunisia; UMR LISAH, Université de Montpellier, INRAE, IRD, Institut Agro Montpellier, AgroParisTech, Montpellier, France; UMR LISAH, Université de Montpellier, INRAE, IRD, Institut Agro Montpellier, AgroParisTech, Montpellier, France; Consultant, Exeter, UK; Hong Kong Baptist University

**Keywords:** Climate change, crop breeding, drought, farming systems, food security, landscape, water use efficiency, WUE

## Abstract

Water scarcity is already set to be one of the main issues of the 21st century, because of competing needs between civil, industrial, and agricultural use. Agriculture is currently the largest user of water, but its share is bound to decrease as societies develop and clearly it needs to become more water efficient. Improving water use efficiency (WUE) at the plant level is important, but translating this at the farm/landscape level presents considerable challenges. As we move up from the scale of cells, organs, and plants to more integrated scales such as plots, fields, farm systems, and landscapes, other factors such as trade-offs need to be considered to try to improve WUE. These include choices of crop variety/species, farm management practices, landscape design, infrastructure development, and ecosystem functions, where human decisions matter. This review is a cross-disciplinary attempt to analyse approaches to addressing WUE at these different scales, including definitions of the metrics of analysis and consideration of trade-offs. The equations we present in this perspectives paper use similar metrics across scales to make them easier to connect and are developed to highlight which levers, at different scales, can improve WUE. We also refer to models operating at these different scales to assess WUE. While our entry point is plants and crops, we scale up the analysis of WUE to farm systems and landscapes.

## Teaser

This review considers approaches to improving water use efficiency beyond the whole-plant level, across scales of time and space, from cells, organs, and plants, to fields, farms, and landscapes.

## Introduction

Agriculture is currently the largest consumer of water. However, as societies develop, the proportion of water use for non-agricultural purposes (industry, domestic) increases, and so does the competition between agriculture and non-agriculture actors. Climate change and its impact on precipitation and increasing temperatures also brings another level of complexity, increasing plant water demand and jeopardizing crop functioning, and indirectly decreasing surface, subsurface, and groundwater resources that supply the crops, whether that be via rainfall or irrigation. Therefore, agriculture in the 21st century needs to become more water efficient, and this goal can only be achieved by considering a holistic approach to water management and use in combination with crop improvement. The purpose of this review is to take that broader look at the functioning of plants and crops and at the multiscale levels of water efficiency, thereby going beyond the usual narrow focus on ‘water use efficiency’ of the plant science community.

Making efficient use of water in agriculture has been the object of much research, and it has been addressed at different scales, with different metrics, and different considerations. In the domain of plant science, ‘water use efficiency’ and ‘transpiration efficiency’ have been the main two metrics, broadly representing a quantity of biomass produced (from units of CO_2_ to grams of biomass) per unit of water used in the wide sense (plant transpiration or crop evapotranspiration that includes both plant transpiration and soil evaporation), and over a time-scale that can vary from sub-seconds to the entire duration of a crop cycle (Farquhar *et al.*, 1982; Condon et al, 2002, 2004; Vadez *et al.*, 2014; Hatfield *et al.*, 2019). The term ‘efficiency’ can also be expanded beyond biomass and be expressed (for example) in units of yield, income, calories, energy, feed value, and protein per unit of water use within the perspective of a farming system, giving a socio-economic angle to the notion of water use efficiency. In the domain of farm engineering, the term ‘irrigation efficiency’ is a common metric that represents the proportion of water (from reservoirs, rivers, and groundwater, for example; often referred to as ‘blue water’) that eventually reaches the roots of the crop (thus becoming ‘green water’, i.e. water contained in the soil profile) and is released back to the atmosphere through transpiration. Beyond tracking and minimizing the water that is lost on the way from the reservoirs to the irrigated fields, increasing irrigation efficiency is also about minimizing the fraction of the water that runs off the fields, gets evaporated, or percolates below the root zone. Whilst the quantity of percolated water is considered as a loss from an agronomical perspective, it is not a loss from a hydrological standpoint since this water infiltrates to recharge the groundwater tables and thus remains present to supply irrigation at the same location or elsewhere, and to fulfil ecosystem services. Finally, it is worth noting that the spatial approach we discuss here needs to be combined with a temporal dimension, where water pathways and subsequent availabilities for crops depend upon seasonal dynamics of both meteorological conditions and agricultural practices.

Therefore, increasing water use efficiency is in part about improving plant water use efficiency *per se* at the leaf, plant, crop, species, agronomy, hydrology, farm, and landscape levels. Beyond this, it is also about maximizing the socio-economic returns from water, not only from a monetary standpoint but also its environmental sustainability with regard to its preservation for future generations. In this review we consider how different research domains collectively address the question of making better use of water in agriculture, providing a broader view on what ‘water use efficiency’ really encompasses, and downscaling its meaning at each disciplinary level. We also aim to find ways, metrics, and equations to connect these scales of analysis. The common theme running through this review is to consider how different traits/crops/plants/anthropogenic actions can contribute to water use efficiency (taken in its broad sense), and whether existing observations and modelling methods can help in connecting these scales.

## Transpiration efficiency at the plant, organ, and cell levels

Many studies aimed at a better understanding of transpiration efficiency (TE) have focused on the organ or cell scales in order to avoid confounding effects of canopy architecture or soil–environment interactions (Vialet-Chabrand *et al.*, 2017; Hatfield *et al.*, 2019; Leakey *et al.*, 2019). At the leaf level, TE is referred to as ‘intrinsic TE’ (TE_int_) and defined as A/G_s_, where A is the CO_2_ assimilation by the photosynthetic biochemistry and G_s_ is the stomatal conductance, or it is also defined as A/T, where T is the water lost by transpiration (see Box 1 for details and further derivations of the ratio). The time-frame is a second or less and the scale is that of a portion of a leaf. Equation 1 posits that possible means by which to increase A/T include increasing C_a_–C_i_ by raising the CO_2_ concentration gradient between the atmosphere and the stomatal chamber, improving the photosynthetic capacity or reducing stomatal conductance, or decreasing W_i_–W_a_ by lowering the water-vapour gradient.

**Box 1. Transpiration efficiency at the leaf scale**

At the leaf level, TE in often called ‘intrinsic transpiration efficiency’ (TE_int_) and defined as A/G_s_, where A is the CO_2_ assimilation by photosynthetic biochemistry and G_s_ is the stomatal conductance, or also as A/T, where T is transpiration. Two equations describing this basic framework have been proposed by Condon *et al.* (2004) and updated by Condon (2020), and are presented here. G_sC_ and G_sW_ are the stomatal conductance for CO_2_ and water, respectively, C_a_ and C_i_ are the CO_2_ concentration in the air and inside the stomatal chamber, respectively, and W_a_ and W_i_ are the vapour pressure in the air and inside the stomatal chamber, respectively. Equation 2 is a simplification of Eqn 1 where the ratio of G_sC_ to G_sW_ is approximated to 0.6 (Condon *et al.*, 2002). Text labels indicate possible levers affecting different terms of the equations.

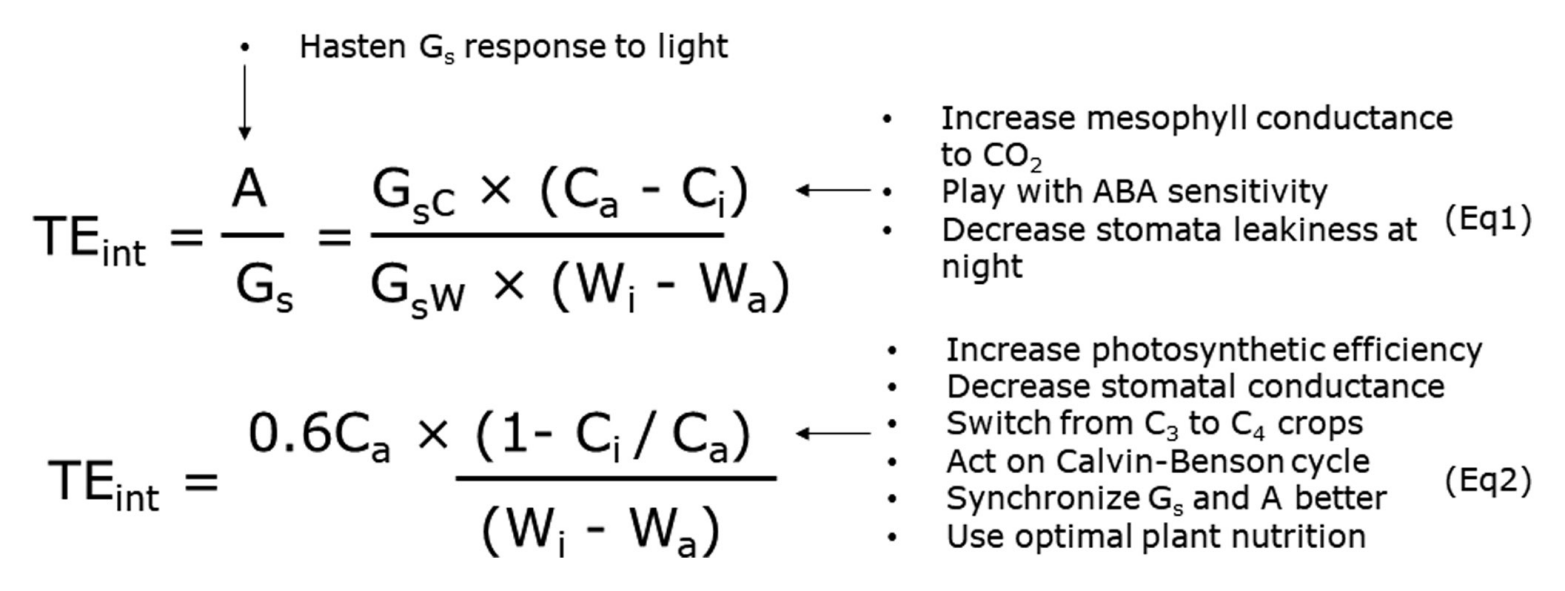

**Table:** 

	Scale/model type	Notes and references
**Time-frame**	< seconds	
**Scale**	Cell, leaf region, organ	
**Models**	G_s_ models	Tardieu *et al.* (2015) Buckley (2017) and Blatt *et al.* (2022) review different approaches for modelling stomatal conductance

Improving TE_int_ by increasing photosynthetic activity is the current object of many studies (Long *et al.*, 2015). In C_3_ plants, an exciting avenue for research is presented by converting their metabolism toward a C_4_-like photosynthesis (Tardieu, 2022); however, inserting C_4_ metabolism in C_3_ plants is a challenging task, as observed in rice (Furbank, 2017; Sedelnikova *et al.*, 2018). A more realistic target might be to concentrate CO_2_ towards the Rubisco active sites by increasing mesophyll conductance to CO_2_, especially in leaves exposed to saturating light. To that end, manipulating the expression of plasma membrane intrinsic protein (PIP) aquaporins that are known to transport CO_2_ from the liquid phase in mesophyll cells is of particular interest (Israel *et al.*, 2021). The expression and functioning of aquaporins have been associated with TE_int_ in rice (Nada *et al.*, 2014) and in the restriction of transpiration during high evaporative demand in soybean (Sadok *et al.*, 2010), pearl millet (Reddy *et al.*, 2017), and chickpea (Sivasakthi *et al.*, 2020). Another promising means for increasing plant photosynthesis is in accelerating the regeneration of ribulose-1:5 bisphosphate RuBP by increasing the levels of photosynthetic enzymes acting in the Calvin–Benson cycle (Long *et al.*, 2015; Simkin *et al.*, 2017) or by stimulating photosynthetic electron transport by overexpressing the Rieske FeS protein that is a key component of the cytochrome *b*_*6*_*f* complex (Ermakova *et al.*, 2019; Simkin, 2019). In tobacco, overexpression of the enzymes fructose-1,6-bisphosphatase/sedoheptulose-1,7-biphosphatase from the Calvin–Benson cycle together with the red algal protein cytochrome *c*_*6*_ serves this purpose and improves TE_int_ and plant biomass under field conditions (López-Calcagno *et al.*, 2020). However, whilst increasing TE_int_ by boosting photosynthetic activity has provided promising results at the cell/organ scale, more efforts are still needed to demonstrate its value in whole plants.

Limiting G_s_ is another option to increase TE_int_, but it may appear less attractive because it might result in a decrease in carbon fixation in the linear parts of the A versus G_s_ relationship (Tardieu, 2022), although it could be interesting in the non-linear parts of the relationship where any further increase in G_s_ is only rewarded by a marginal increase in A. Nevertheless, it is usually assumed that strategies aimed at reducing G_s_ come at the expense of biomass production and yield under optimal conditions (Condon *et al.*, 2002; Blum, 2009; Roche, 2015), and that they might be more useful under water limitation (Hughes *et al.*, 2017; Caine *et al.*, 2019; Mega *et al.*, 2019). However, this assumption is being challenged by an increasing number of reports showing that limiting G_s_ might not necessarily lead to a decrease in A (Franks *et al.*, 2015; Yang *et al.*, 2016; Dunn *et al.*, 2019). For instance, robust and large-scale lysimetric assessments of pearl millet, sorghum, and groundnut have challenged the common view that higher transpiration efficiency is bound to lower productivity, and have shown that higher TE is completely unrelated to total plant water use, which in turn is directly related to plant productivity (Vadez *et al.*, 2014). In another example, transgenic wheat plants overexpressing *Epidermal Patterning Factor* (*EPF*) show an increase in TE_int_ with no changes in A, biomass, and yield compared to control plants when the reduced stomatal density is no more than 50% of that of the controls (Dunn *et al.*, 2019). Transgenic tomato plants overexpressing 9-cis-epoxycarotenoid-dyoxygenase have higher ABA than the wild-type and show higher TE_int_ because of a lower stomatal conductance (Thompson *et al.*, 2007). Decreasing G_s_ by increasing plant sensitivity to ABA via overexpression of the ABA receptor *REGULATORY COMPONENT OF ABA RECEPOR 6* (*RCAR6*) in Arabidopsis also results in an unexpected increase in A and in a higher TE_int_ (Yang *et al.*, 2016). It is still unclear whether this ABA-related effect on A is the result of an increase in mesophyll conductance to CO_2_, greater Rubisco activity, or is due to other pleiotropic aspects related to the effects of ABA on leaf characteristics, such as stomatal density or leaf epinasty, which could improve radiation interception (Thompson *et al.*, 2007; Yang *et al.*, 2016; Condon, 2020). Another interesting example comes from the overexpression in tobacco of *PHOTOSYSTEM II SUBUNIT S* (*PsbS*), which encodes a protein stimulating the non-photochemical quenching that protects the photosynthetic machinery under excessive light (Głowacka *et al.*, 2018). PsbS promotes thermal dissipation of excitation energy under high light and keeps the redox state of chloroplastic QUINONE A more oxidized, with the latter protein being an early signal for stomatal opening when it is reduced. Plants with increased *PsbS* expression growing in field conditions show increased non-photochemical quenching and lower G_s_ in response to light, resulting in a 25% reduction in water loss per CO_2_ assimilated (Głowacka *et al.*, 2018). Limiting night transpiration by limiting G_s_ under dark conditions also contributes to the increase in TE (Coupel-Ledru *et al.*, 2016; Fricke, 2019).

The dynamic/temporal responses of G_s_ to environmental conditions have emerged as a novel approach in improving TE_int_. In the field, fluctuations in light intensity and spectral quality that influence the photosynthetic photon flux density (PPFD) in term have large effects on A and G_s_ (Way *et al.*, 2012). However, stomatal responses are an order of magnitude slower than photosynthetic responses (minutes versus seconds), which leads to a disconnection between G_s_ and A. This relative lag in G_s_ limits A as stomata are slow to open under increasing PPFD, whilst unnecessary water loss continues after A has dropped under decreasing PPFD (Vialet-Chabrand *et al.*, 2017). Simulations suggest that synchronizing the behavior of G_s_ and A could increase TE_int_ by 20% in *Phaseolus vulgaris* under fluctuating PPFD (Lawson *et al.*, 2014). Manipulating stomatal movements to reduce the G_s_ response time and to improve water use and growth have been achieved in Arabidopsis by overexpressing *BLUE LIGHT-GATED K*^*+*^*CHANNEL 1* (*BLINK1*) specifically in guard cells (Papanatsiou *et al.*, 2019). The mean half-times of stomatal opening and closing upon exposure to light and dark, respectively, were accelerated by ~40% compared with the control plants, resulting in a 2.2-fold increase in biomass under fluctuating light without a cost in water use by the plant, thus increasing TE. Interestingly, large variations have been observed for A and G_s_ both among species (13 species varying in the shape of the stomata guard cells; McAusland *et al.*, 2016) and within species (in wheat, Salter *et al.*, 2019; in sorghum, Pignon *et al.*, 2021), and this has the potential to be exploited.

In summary, contrary to common belief, there may be several options to increase TE by adjusting G_s_ without significantly altering A, which would allow the development of water-efficient cultivars without significant yield trade-offs.

## Transpiration efficiency at the plant and crop levels: interactions with the soil and atmosphere

Transpiration efficiency (TE) at this scale is measured in grams of biomass dry weight produced per unit of water transpired (B_d_/T, g biomass l^–1^), not taking into account soil evaporation. Sinclair *et al.* (1984) initially expressed TE as k_d_/${\left(\bar{e_{a}^{*}-e}\right)}_{d}$ , where ${\left(e_{a}^{*}e\right)}_{d}$ is the gradient in vapour pressure between the leaf and the atmosphere at air temperature and the denominator represents a daily mean, and k_d_ is akin to the numerator term of Eqn 2 that reflects the C_i_/C_a_ ratio (Box 1). In Box 2, we consider how to increase TE via the denominator term, ${\left(e_{a}^{*}e\right)}_{d}$, which is putatively an environmental factor, looking at daily or seasonal time-scales. The definition of TE implies that it will increase when the integration of the denominator over time is small. At a daily time-scale, this would mean avoiding transpiration during hours of the day with the highest vapour pressure deficit (VPD). At the time-scale of a crop season, avoiding periods with high VPD conditions would have the same effect, for example by early sowing. Although the numerator term is considered as constant for C_3_ and C_4_ species (4 Pa and 9 Pa respectively; Sinclair *et al.*, 1984), variations in TE among C_4_ species have been found (Vadez *et al.*, 2021), which implies variations in the k_d_ term since the experiments were carried out side by side. Measuring TE is difficult, especially in the field (Cooper *et al.*, 1983), as it requires precise transpiration and biomass measurements, although a lysimetric method has managed to reconcile precision and throughput (Vadez *et al.*, 2014). These authors reported large variations in TE among panels of cultivated germplasms of sorghum, pearl millet, and groundnut. Many studies have used ^13^C discrimination as a proxy for TE, although a number of them have reported limits to the value of this method (see Vadez *et al.*, 2014, for a detailed discussion and references).

Crop simulation has shown that restricting transpiration under high VPD can increase TE (Sinclair *et al.*, 2005). For the plant, this process translates into opening stomata and maximizing A when W_i_–W_a_ is low due to low VPD in the air (Box 1) and closing stomata when VPD exceeds a certain threshold (Sinclair *et al.*, 2017). Stomatal closure under high VPD will limit A and increase leaf temperature because of decreased evaporative cooling, but since it is restricted to a period of a few hours, it is expected to avoid an excessive trade-off in terms of CO_2_ fixation. Integrated over the time-course of a day (e.g. *n*=12 h; Box 2), the denominator term would exclude the high vapor pressure gradients of the midday hours, and thus increase TE. A similar integration could be carried out over the time-scale of a crop season, for example *n*=120 d. Clear agronomical benefits of phenotypes that restrict transpiration under high VPD in water-limited conditions have been demonstrated in maize (Messina *et al.*, 2015) and sorghum (Kholová *et al.*, 2014). Experimental evidence of genotypic variation for this trait has been reported in soybean (Fletcher *et al.*, 2007), pearl millet (Kholova *et al.*, 2010), chickpea (Zaman-Allah *et al.*, 2011), sorghum (Choudhary *et al.*, 2014), and wheat (Schoppach *et al.*, 2012).

**Box 2. Transpiration efficiency at the plant scale**

Equation 3 defines TE as the ratio of biomass (B_d_) to transpiration (T) and builds on an earlier equation (Sinclair *et al.*, 1984), that gives a daily TE value such as TE = k_d_/${\left(e_{a}^{*}e\right)}_{d}$, where e_a_* is the saturation vapour pressure at air temperature, e is the vapour pressure in the air, the denominator ${\left(e_{a}^{*}e\right)}_{d}$represents a daily mean, and the term k_d_ is a factor that reflects the CO_2_ concentration in the stomatal chamber, i.e. the C_i_/C_a_ term of Eqn 2 in Box 1 (Condon *et al.*, 2002). The term (e_a_*–e)_d_ then represents water vapor pressure deficit (VPD). In Eqns 3 and 4 below, the integration is conducted at the time-scale of days (d) to give a mean value of TE over i=1 to i=*d* days, and clearly other periods can be considered, such as hours. Text labels indicate possible levers either directly or indirectly affecting the terms of the equations.

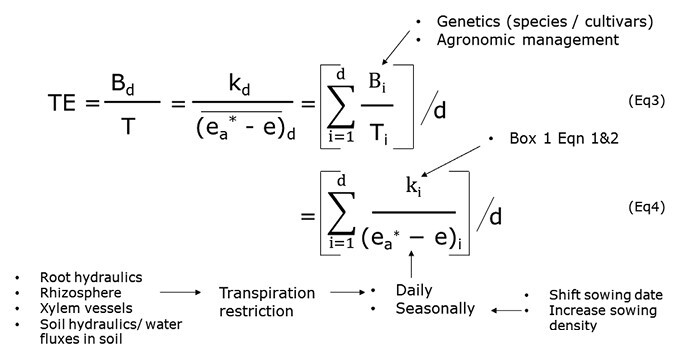

**Table:** 

	Scale/model type	Notes, references, and links
**Time-frame**	Day(s) to months	Up to a crop cycle
**Scale**	Plant, crop	
**Models**	Functional–structural plant models (FSPM). Crop models in Box 3	CPlantBox (Zhou *et al.*, 2020); https://www.quantitative-plant.org/model/cplantbox

As noted above, the first benefit of restricting transpiration under high VPD is to increase TE, and this has been demonstrated in sorghum lines introgressed with the *staygreen* QTL (Vadez *et al.*, 2011), which restricts transpiration under high VPD. This study was followed by a modelling assessment of the benefit of the restriction of transpiration on the yield in the same lines (Kholová *et al.*, 2014). In maize, some lines with relatively high TE more tightly regulate their transpiration response to increases in VPD and have a change-point at lower VPD levels than lines with lower TE (Ryan *et al.*, 2016). As far as we know, there have been no other experimental reports linking a milder transpiration response to VPD with higher TE, and additional experimental evidence is needed. Notably, pearl millet genotypes contrasting in their transpiration responses to high VPD (Kholova *et al.*, 2010) do not differ in terms of TE (Vadez *et al.*, 2013), suggesting that restriction of transpiration does not always increase TE. The interpretation given by Kholova *et al.* (2010) is that the observed restriction of transpiration could result from a mix of fully closed and fully open stomata, giving no benefit in intrinsic TE for the open stomata and yet still reducing the overall transpiration because of the closed stomata.

The second, and possibly most important benefit of the restriction of transpiration under high VPD comes from more parsimonious water use at early stages in the life cycle, which subsequently makes more water available to plants for the critical grain-filling stage. This has been shown in a number of crops such as pearl millet (Vadez *et al.*, 2013, 2014), chickpea (Zaman-Allah *et al.*, 2011), and maize (Messina *et al.*, 2015). Similar responses have been observed in modern Spanish durum wheat lines grown under drought stress in field conditions (Medina *et al.*, 2019). Water availability during the grain-filling period is indeed critical, and it has been shown to have a high return in terms of grain yield per mm of water, for example 55 kg ha^–1^ mm^–1^ in wheat (Manschadi *et al.*, 2006), ~40 kg ha^–1^ mm^–1^ in chickpea (Zaman-Allah *et al.*, 2011), and 37–45 kg ha^–1^ mm^–1^ in pearl millet (Vadez *et al.*, 2013). This also tells us that improving transpiration ‘efficiency’ is not only about improving the physiology of plant transpiration (Box 1) but also about understanding time periods when the crop has critical water needs (Box 3). In that sense, the equation Yield = T×TE×HI, where T is water used for transpiration and HI is the harvest index (Passioura, 1977), can no longer be seen as the combination of linear terms, but as the combination of terms whose importance varies among them and over time (see Box 3, and below).

**Box 3. WUE at the crop/field scale**

At this scale transpiration efficiency is generally termed water use efficiency (WUE_field_) and is the ratio of grain yield to water used, either coming from rainfall or irrigation (Eqn 5). Yield can be disaggregated in the equation Yield = T × TE × HI (Passioura, 1977), where HI is the harvest index, and T and TE are brought in from Box 2. In Eqn 6, this ratio is reduced into a sum of daily ratios of TE to the proportions of water lost to evaporation and to run-off, which is then integrated over a season of n days. This equation indicates that soil evaporation and run-off need to be minimized to maximize WUE. Text labels indicate possible levers directly affecting the terms of the equations.

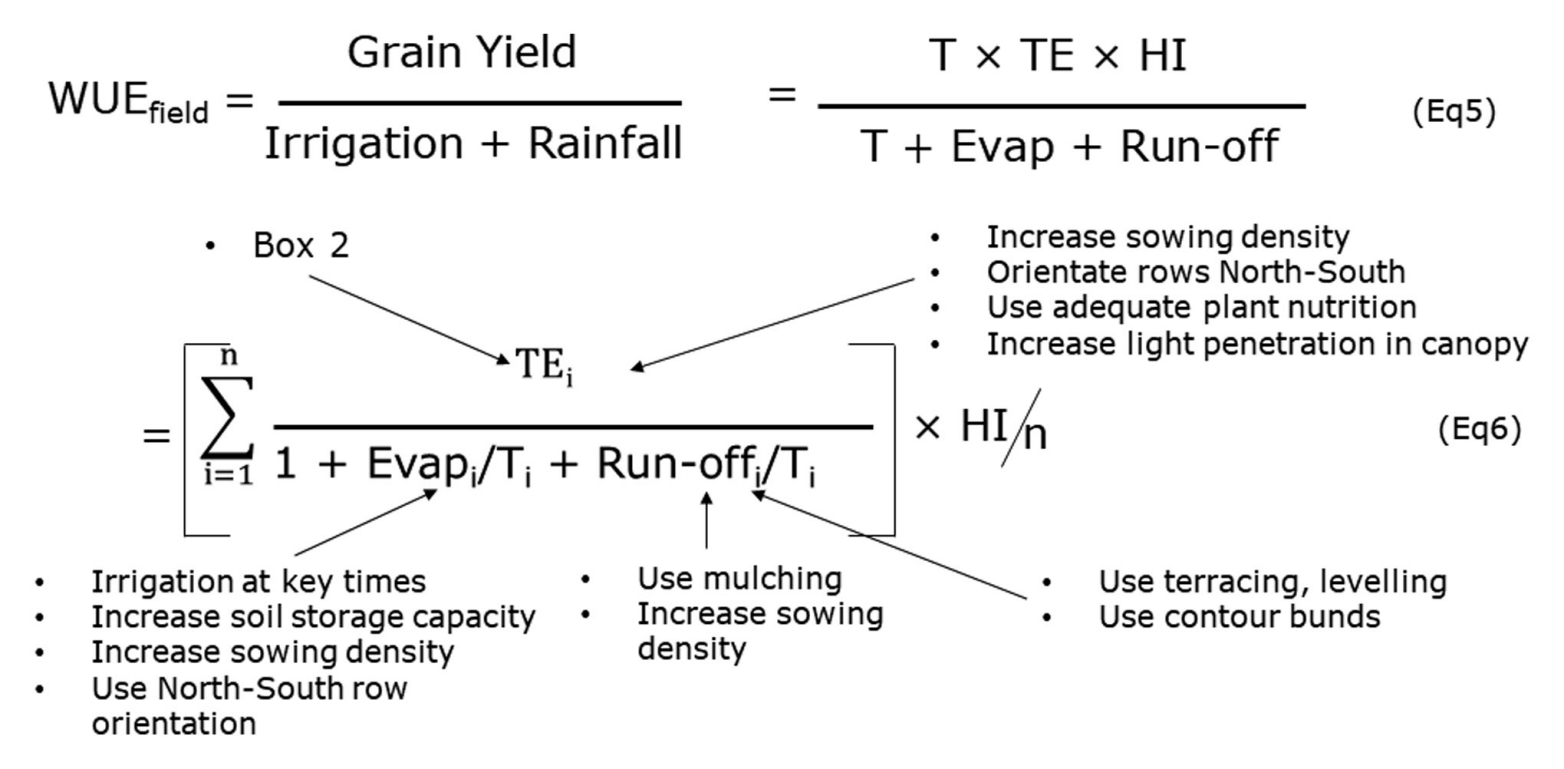

**Table:** 

	Scale/model type	Notes, references, and links
**Time-frame**	Months	Crop cycle
**Scale**	Crop, field	
**Models**	Process-based crop simulation models such as: APSIM; DSSAT; WOFOST; DAISY; CROPSYST; AQUACROP; SSM	https://www.apsim.info/ https://dssat.net/ https://www.wur.nl/en/research-results/research-institutes/environmental-research/facilities-tools/software-models-and-databases/wofost.htm https://daisy.ku.dk/ http://sites.bsyse.wsu.edu/cs_suite/cropsyst/ https://www.fao.org/aquacrop/en/ http://ssm-crop-models.net/

Late-night transpiration, as was reported in wheat (Tamang *et al.*, 2019), has been proposed as an avenue for research towards increasing TE, on the assumption that it would set the plant for an early onset of photosynthesis in the morning under more favorable VPD conditions, possibly related to a higher pre-dawn water status. That said, night-time transpiration is not associated with any photosynthesis and would need to be limited. Up to 30% of plant water loss can take place at night in grapevines (Coupel-Ledru *et al.*, 2016) and up to 55% in wheat (Schoppach *et al.*, 2014), so that reducing night-time transpiration would be a way of improving TE.

### What drives the restriction of transpiration?

According to the gradients in potential, water moves from the soil pores to the roots, and then through the plant to the stomatal chamber to be transpired as vapour into the air (Tyree, 1997). Hydraulic resistances have been identified at different levels in this continuum, affecting the water gradient between the soil and the plant, gradients within the plant, and between the plant and the air. Typically, resistances across the soil, across the soil–root interface, across the root to the root xylem, and along the xylem are used to model water flow through a series of analytical solutions/functions (e.g Couvreur *et al.*, 2012; Abdalla *et al.*, 2022; Koehler *et al.*, 2022). Accurate estimates of how soil water stress affects plant transpiration are essential for reliable mechanistic model predictions (Verhoef *et al.*, 2014) so that reliable exploration of possible effects on TE can be carried out. There is still a need for such estimates, and models that can easily represent mesophyll behavior in response to environmental drivers are still rare (Zhang *et al.*, 2022).

Soil hydraulic conductivity can drastically limit water uptake and is considered as a main driver of stomatal closure for plants in drying soils (Carminati *et al.*, 2020; Carminati and Javaux, 2020; Abdalla *et al.*, 2022). Its effects can interact with crop species/genotypes. As an illustration, maize and sorghum genotypes have been observed to have lower TE in a sandy soil than in a high clay Vertisol, whereas TE is similar in both soils in pearl millet (Vadez *et al.*, 2021). It has been proposed that species fitness could be specific to soil type and its interaction with the environment (low/high VPD). Plants respond to soil matric potential (suction) rather than soil water content (SWC), a concept that has long been understood and is widely accepted. The magnitude of matric potential depends on SWC, the size of the soil pores, the surface properties of the soil particles, and the surface tension of the soil water (Whalley *et al.*, 2013). Thus, in summary, a putative role of soil in possibly explaining restrictions of transpiration needs to be clarified (Box 2).

Experimental evidence further suggests that root phenotypes such as long and dense root hairs postpone soil limitation in drying soils by reducing the drop in matric potential at the interface between the roots and soil in transpiring plants (Carminati *et al.*, 2017; Cai *et al.*, 2022; Schnepf *et al.*, 2022). The nature of the rhizosphere also has the potential to disrupt the connection between the soil and the plant. Engineering rhizospheric characteristics, for example by increasing mucilage production, might open up new avenues for crop production management and lead to increases in water use efficiency (Ahmed *et al.*, 2018). In addition to their role in water capture, roots have been proposed to act as hydraulic rheostats, able to adjust their hydraulic radial conductance through alterations of apoplastic barriers (Calvo-Polanco *et al.*, 2021; Salas-González *et al.*, 2021) or aquaporin functions (Maurel *et al.*, 2010; Vadez, 2014). A typical example comes from the correspondence observed between aquaporin expression, diurnal variations in root hydraulic conductivity, and transpiration, which can be interpreted as a means for preventing a drop in water potential in the leaf when transpiration is high (Tsuda *et al.*, 2000). Restriction of transpiration under high VPD has indeed been related to root conductance and its control by root and shoot aquaporins (Sadok *et al.*, 2010; Reddy *et al.*, 2017, 2022; Sivasakthi *et al.*, 2020), but also to leaf area (Choudhary *et al.*, 2020) and to the root-to-shoot ratio (Affortit *et al.*, 2022). Xylem vessels are responsible for hydraulic axial conductance of water from the roots to the shoots, and reductions in xylem conductance have been associated with increases in TE in wheat (Richards *et al.*, 1989; Hendel *et al.*, 2021).

In summary, there are two main benefits to restricting transpiration under high VPD, namely higher TE and more water for grain filling. Several root traits and soil characteristics are likely to have a strong influence on the restriction, and will tend to decrease the denominator term of Eqn 4 (Box 2) and thereby increase TE overall.

## Water use efficiency at the species and agronomy level

This scale of assessment is at the level of field plots, and transpiration efficiency is generally referred to as water use efficiency (WUE_field_). The usual metric is either grain or biomass yield per millimeter of water used (kg ha^–1^ mm^–1^) from either rainfall or irrigation (Box 3). For an easier connection to the other scales, WUE_field_ can also be expressed with the metrics presented above (Box 2), by converting yield into the product of plant transpiration T, TE, and the harvest index (HI; Passioura, 1977), and by separating rainfall and irrigation into the transpiration component T minus a component of soil evaporation and run-off (Eqn 5, Box 3). Equation 6 is then a daily integration over an entire crop cycle, following the integration developed in Box 2. As a result, it becomes clear that increasing WUE_field_ is about increasing transpiration efficiency (as detailed in the previous two sections) *and* minimizing soil evaporation and run-off.

Run-off occurs when rainfall (more rarely irrigation) is in excess of what the soil can absorb. The term ‘precipitation use efficiency’ (PUE) can be used and is an integration of the yield increments that occur consecutive to any rainfall. Research on the capacity of soil to store more water has been aimed at improving PUE under such rain-fed conditions (Hatfield *et al.*, 2001). For example, over-tilling of bare soil leads to decreases in its water- and nutrient-storage capacities that in turn decrease the potential WUE of the future crop (Cresswell *et al.*, 1993). A scale-up of PUE is precipitation storage efficiency (PSE), which defines the capacity of a soil to be a more or less useful reserve for future crops. PSE is negatively affected by over-tilling (Tanaka *et al.*, 1987) and it has been reported that it can be increased by up to 40% by using herbicides to control weeds instead of conventional tillage (Wicks, 1968). It has been demonstrated that PSE, PUE, and WUE are related (Nielsen *et al.*, 2005) (Box 3), and thus soil management practices can potentially be used to increase WUE.

As far as soil evaporation is concerned, early vigor is a plant trait that has long been favored by breeders, as it ensures rapid coverage of the ground and efficient competition against weeds. A faster soil coverage could also come from an increased sowing density, and this would reduce the evaporation component of the equations for WUE_field_ (Box 3). There are also promising avenues to explore for decreasing irrigation needs and improving WUE_field_ in semi-arid regions by using advanced agronomic practices such as those related to conservation tillage (DeLaune *et al.*, 2012) and mulching (Igbadun *et al.*, 2012; Liao *et al.*, 2021), which are considered as effective means for improving irrigation efficiency by reducing the fraction of water lost through non-beneficial soil evaporation. They can also allow for the control of weeds, reduction of soil compaction, improvements in nutrient management, and the incorporation of additional nutrients into the soil (McCraw *et al.*, 1991; Shaxson *et al.*, 2003). It has been reported that plastic film and straw mulching also reduces the impact of raindrops on the soil surface, subsequently reducing soil dispersion and thereby enhancing water infiltration, reducing run-off, and increasing soil water storage (Li *et al.*, 2013).

As far as the TE component of Eqn 6 is concerned (Box 3), the WUE_field_ of crops can be affected by the management applied and be optimized by the right interaction of genotype and management (G×M) for a given environment (E) (Hsiao *et al.*, 2007; Messina *et al.*, 2009). In pearl millet, low soil-P treatments have been reported to decrease WUE (Beggi *et al.*, 2015), and it has been shown that both the intrinsic TE (A/T, Box 1) and TE at the plant level (B_i_/T, Box 2) are decreased by ~10-fold by a low soil-P treatment (Payne *et al.*, 1992). It should be noted that low P nutrition has also been shown to reduce plant hydraulic conductance (Radin, 1990). A number of studies also show increased WUE as soil fertility increases (e.g. in wheat, Fan *et al.*, 2005; and in maize, Faloye *et al.*, 2019). Therefore, poor fertility is bound to decrease the TE component of Box 3, something that is food for thought when agriculture needs to be more water-efficient while also aiming at using less nutrients. Increasing the sowing density in maize has been shown to increase WUE (French and Schultz, 1984; Hatfield *et al.*, 2001), and is interpreted as an effect of a limited leaf area index. In cotton, a higher sowing density was found to affects the microclimate within the canopy, with light transmission through the canopy in some genotypes increasing light interception (Yang *et al.*, 2014). As an alternative interpretation, the benefit could also have been the result of a combination with a decrease in VPD within the canopy (Box 2), allowing photosynthesis to continue at the lower VPD, and hence achieving higher gains in intrinsic TE_int_ (Box 1). Indeed, recent work on sorghum has highlighted a significant increase in WUE when the sowing density of plants is doubled, with large genotypic variation being found in the response (Pilloni, 2022). This can be contrasted with a lower WUE found in a skip-row planting system and dry environment, and a higher WUE found in a skip-row system and a wetter environment (Abunyewa *et al.*, 2010). In the dense-canopy conditions used by Pilloni (2022), high WUE was recorded in genotypes with a strong transpiration response to the evaporative demand, which was seemingly caused by a higher light penetration within the canopy. The benefit thereby came from the association of an agronomic management modification (density) and a genetic trait related to canopy architecture that allowed light penetration within the canopy. The plant architectural traits seem to have been driving the diversity in the response. According to Niinemets (2010), the traits that most control the distribution and efficiency of light use at the plant level are the angle distribution of the leaves and the spatial aggregation of the foliage. From a canopy perspective, the way leaves are spatially distributed and how biomass is allocated to them varies significantly. Unfortunately, leaf areas are still largely represented using 2D metrics (m^2^), and more work is needed to better understand and measure leaf areas in 3D, and hence to better gauge the role they play in light distribution through the canopy and what effects they have on the microclimate within the canopy.

### WUE can be linked to crop architecture, orientation, and associations with other plants–

In the previous section we have shown that adapting the management of an annual crop can directly affect its water budget. The same is true for perennial crops, but in this case the management also needs to be adapted to the landscape. A striking example is that of the vineyard, where the orientation of the rows of plants affects how the soil temperature and water content vary with depth (Hunter *et al.*, 2020), and this can have an impact on water availability at the scale of the landscape. The main factor that is affected by row orientation in perennial crops is the distribution of light resources through the canopy. A change in orientation in a Shiraz vineyard from north–south to east–west was found to significantly reduce the transpiration by up to 13% without any significant reduction in yield and its components, thus leading to an increase in WUE (Buesa *et al.*, 2017). It was suggested that the better distribution of radiation received by the rows orientated east–west during the hot and dry season and the low photosynthetic efficiency of the north–south vines during the afternoon contributed to the increased WUE.

These differences in canopy structure and the link to WUE can be also considered in the case of intercropping between annuals or between perennials and annuals. For example, maize yield in a maize–coffee tree intercropping system has been found to be increased by 50–80%, depending on the intercropping distance, compared to maize grown alone (Huxley *et al.*, 1994). This was possibly due to lower direct radiation, a milder microclimate effect due to tree transpiration, and lower exposure of the maize to wind. Reduced exposure to wind would decrease the evaporative demand to the benefit of WUE, and ultimately yield (Chaves *et al.*, 2016; Hatfield *et al.*, 2019). These case studies show the importance of planning combinations of crops within agroecosystems that can best use the different resources and tolerate the environmental constraints that drive the pattern of water consumption (see landscape section below).

### Increasing WUE through better irrigation efficiency

Irrigation efficiency (IE), defined as the ratio of water used by the crop for transpiration to total water applied, is the traditional concept of efficiency in irrigation engineering (Israelson, 1950, Jensen, 2007). Equation 7 in Box 4 separates irrigation into the components directed to crop transpiration, soil evaporation, run-off (similar to Box 3), and percolation below the root zone.

**Box 4. Increasing WUE at the field scale with better irrigation efficiency**

Irrigation efficiency (IE) represents the proportion of irrigated water that will eventually be used for plant transpiration and hence for growth. Here, Eqns 7 and 8 only focus here on the ‘T’ component from the previous Boxes 1–3. The integration also in Eqn 8 is done over a season of n days. Equation 7 introduces a component of water percolation below the root zone (Perc). Although they are not represented in the equations, water losses can also occur during the transfer from the source to the field, or during the application of irrigation. This can be measured as the conveyance efficiency, defined as the ratio of water diverted from the source (reservoir, river, pumping station) to the water reaching the field (Rogers *et al.*, 1997; Howell, 2003), and as the field application efficiency, defined as the ratio of water needed by the crop to the amount of water available at the field inlet (Bos and Nugteren, 1990). Text labels indicate possible levers directly affecting the terms of the equations. DI, deficit irrigation; RDI, regulated deficit irrigation; PRD, partial root-zone drying.

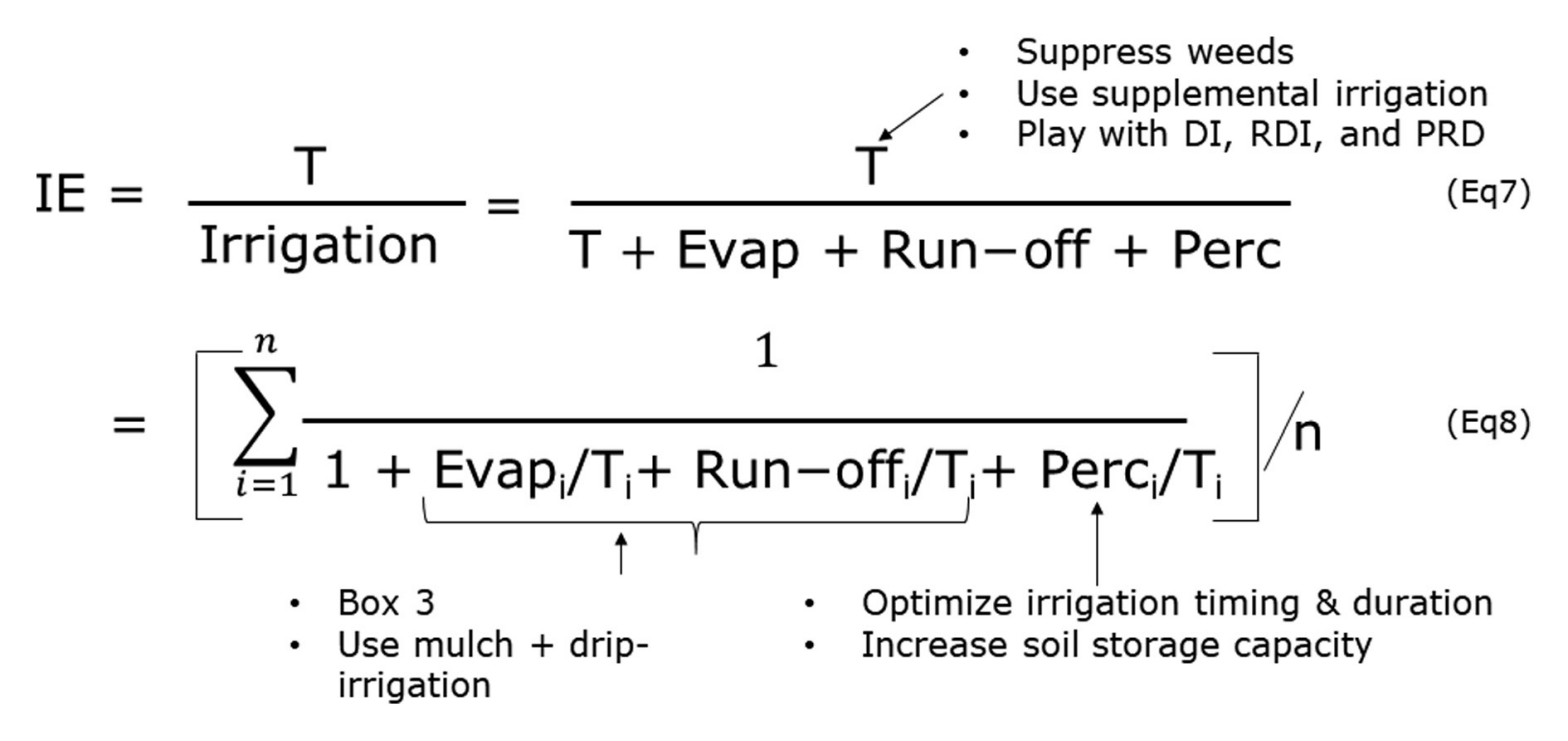

**Table:** 

	Scale/model type	Notes, references, and links
**Time-frame**	Months	Crop cycle, years
**Scale**	Field, irrigation basin	
**Models**	Crop models of Box 3. Irrigation management models such as:HYDRUS; CROPWAT; or other Agrohydrology models such as Soil Water Atmosphere Plant (SWAP)	https://www.pc-progress.com/en/default.aspx?hydrus https://www.fao.org/land-water/databases-and-software/cropwat/en/ https://www.swap.alterra.nl/

Improvement of IE at the field level can be achieved by reducing evapotranspiration from weeds, and by adopting practices such as optimizing the timing of irrigation, reducing waterlogging, and using advanced irrigation techniques to reduce the wetted area (Batchelor *et al.*, 2014; Hatfield *et al.*, 2019). A series of new irrigation practices and technologies have been developed to enhance WUE based on the physiological mechanisms of crop responses to water deficit. These irrigation strategies encompass deficit irrigation, regulated deficit irrigation, and partial root-zone drying irrigation, and can be applied by surface-, sprinkler-, or drip-irrigation methods, or possibly by sub-surface methods to avoid soil evaporation (El-Hendawy *et al.*, 2008; Jovanovic *et al.*, 2020).

Deficit irrigation (DI) is a water-saving strategy that permits a certain level of crop water stress to exist continuously throughout the season without compromising crop yield significantly (Pereira *et al.*, 2002; Manning *et al.*, 2018). Regulated deficit irrigation (RDI) allows water stress at certain phenological stages during which plants are less sensitive, while fully meeting the irrigation needs of the crop at critical growth stages (Romero *et al.*, 2013). Partial root-zone drying (PRD) irrigation is a technique that allows half of the root system to experience drying while the other half is irrigated. PRD targets the plant physiological response through the production of abscisic acid (ABA) by the drying roots, which reduces leaf expansion and stomatal conductance (thus affecting C_i_/C_a_ in Box 1) while the wetted roots maintain a favorable plant water status (Galindo *et al.*, 2018). It has been reported that RDI and PRD improve WUE mainly through enhancing the guard-cell signal transduction network that reduces leaf transpiration (Schroeder *et al.*, 2001), through optimized stomatal control that improves the ratio of photosynthesis to transpiration (Iqbal *et al.*, 2020), and through a reduction in the evaporative surface area (Xie *et al.*, 2012). However, there is still debate about the importance of ABA signalling in regulating WUE in plants subjected to PRD (e.g. Perez-Perez *et al.*, 2012) because ABA production in the part of the root exposed to drying might not be sustained over time (Dodd *et al.*, 2008).

In rain-fed agriculture of semi-arid regions, supplemental irrigation (SI) has emerged as a promising practice for climate resilience. It consists of applying limited amounts of water at critical growth stages when rainfall fails to provide sufficient moisture for normal crop growth in order to improve and stabilize yields (Oweis *et al.*, 2012) (Box 4). Several studies have reported substantial increases in crop yields using this method. For example, Oweis *et al.* (2000) showed that, in combination with early sowing and the availability of appropriate levels of nitrogen, the WUE of rain-fed wheat can be substantially improved by adopting a level of SI equivalent to only one-third to two-thirds of the full irrigation requirement. Timely SI at the jointing and anthesis growth stages of wheat can result in high grain yields and nitrogen use efficiency while achieving higher WUE (Wu *et al.*, 2018), and chickpea yield can be increased 30% by applying 40 mm irrigation at the beginning of seed growth (Vadez *et al.*, 2011).

Finally, crop management and irrigation techniques can be combined to further increase irrigation efficiency. In a modelling study that analysed the effects of mulching and drip-irrigation, Zhang *et al.* (2022) found that a combination of the two treatments significantly improved the crop yield and WUE compared to irrigation with no mulching. The improvements were affected by climatic and soil conditions, crop type, and water consumption, with the technique being more effective in planting areas with rainfall or water consumption less than 400 mm and in areas with soil of medium texture.

## Maximizing WUE at the farm system level

Water efficiency at the farm level is often calculated as the ratio of total farm production to the total amount of irrigation water used. In economic terms the word ‘production’ usually means the gross margin of a farm (revenue minus production costs), and so this efficiency can also be expressed in terms of the number of calories produced by the crops grown on the farm, and/or by the negative or positive externalities that a farm can produce, for example the quantity of water used, nitrogen leached, changes in soil organic matter, erosion, and drainage.

Efficiency is often considered on a per-hectare basis for easy comparisons at the regional level. To better understand this overall efficiency, it is often necessary to calculate and analyse intermediate efficiencies. These may be expressed by type of crop, crop practice, irrigation system, or biophysical system. The concept of water efficiency at the farm level is rarely used for rain-fed crops. Finally, the concept of irrigation efficiency at the farm level is often used to characterise and analyse the performance of farms with regard to the quantities of irrigation water involved, to put forward and test the effects of technical (e.g. new varieties, new rotation, tillage) and socio-economic (e.g. water pricing, subsidies for more efficient irrigation systems) alternatives meant to improve water efficiency and consequently the overall performance of the farm, or to better understand the determinants that affect overall water efficiency (soil effects, rotation, irrigation systems).

### The concept of water efficiency at the farm level

The stochastic frontier production approach is the most widely used method to analyse technical efficiency in production (Battesse *et al.*, 1995). Irrigation water technical efficiency (IWTE) measures how an individual farmer’s water use compares with that of the most efficient water user. The comparison is made while controlling for the effects of all other factors affecting efficiency (Yigezu *et al.*, 2013). IWTE_i_, where ‘i’ represents a farm, is calculated following Karagiannis *et al.*, (2003) (Box 5). This efficiency formulation is based on three considerable simplifications: all production factors other than water are less limiting, all these factors act in the same way on production, and all these production factors are substitutable. For this reason, these factors are often expressed in monetary terms for easier aggregation, such as production costs, and not in quantitative terms; in this case, we refer to irrigation technical cost efficiency, ITCE (Akridge, 1989) (Eqn 10, Box 5). This formulation implies that improving farm efficiency can come from acting either on the denominator (e.g. switching from flooding to drip irrigation), the numerator (e.g. improving crop yields by using more efficient varieties), or both. In practice this concept is complicated to apply, especially in an arid context where several limiting factors act at the same time (e.g. labour, access to resources and the market) and where several production objectives are targeted (e.g. food, economic, social, environmental). In this context, comparing and especially understanding the efficiency of a farm in relation to water involves verifying that no other factor is limiting, and that water is the only determining factor in the total performance of the system being analysed. Here, the main issue in calculating efficiency is not the mathematical formulation of efficiency, but the availability and quality of data (see Appendix S1 for more details).

**Box 5. WUE at the farm scale**

Irrigation water technical efficiency (IWTE) measures how an individual farmer’s water use compares with that of the most efficient water user. IWTE_i_, where ‘i’ represents a farm, is determined according to Eqn 9, where X_1_ represents the units of inputs other than water, W_2_ represents the minimum feasible water use needed to produce the optimal units of output, C, and A represents the quantity of non-optimal units of output that would be obtained from the same level of X_1_ combined with a non-optimal quantity of irrigation water, W_2_. The ratio W_1_/W_2_ expresses the proportion of irrigation water that is lost, or alternatively the proportion saved [1–( W_1_/W_2_)]. It also enables the determination of the maximum possible reduction in water use (W_1_−W_2_). If water is not available at the right time, this leads to a reduction in the denominator in Eqn 9 (Box 9; quantity of irrigation water, W_2_) but a fairly small increase in the numerator, mostly expressed by the gross margin on the farm. The irrigation technical cost efficiency (ITCE) is defined for the *i*th studied farm (ITCE_i_) in Eqn 10, where S_W,i_ is the observed share of the cost of irrigation water (W) out of the total of all input costs of the *i*th studied farm, and S_j,i_ is the corresponding share of the cost of the *j*th input. By definition, the shares of the costs of all the inputs must add up to 1 and since IWTE_i_ takes values between 0 and 1 (Eqn 9), it implies that ITCEi is the same.

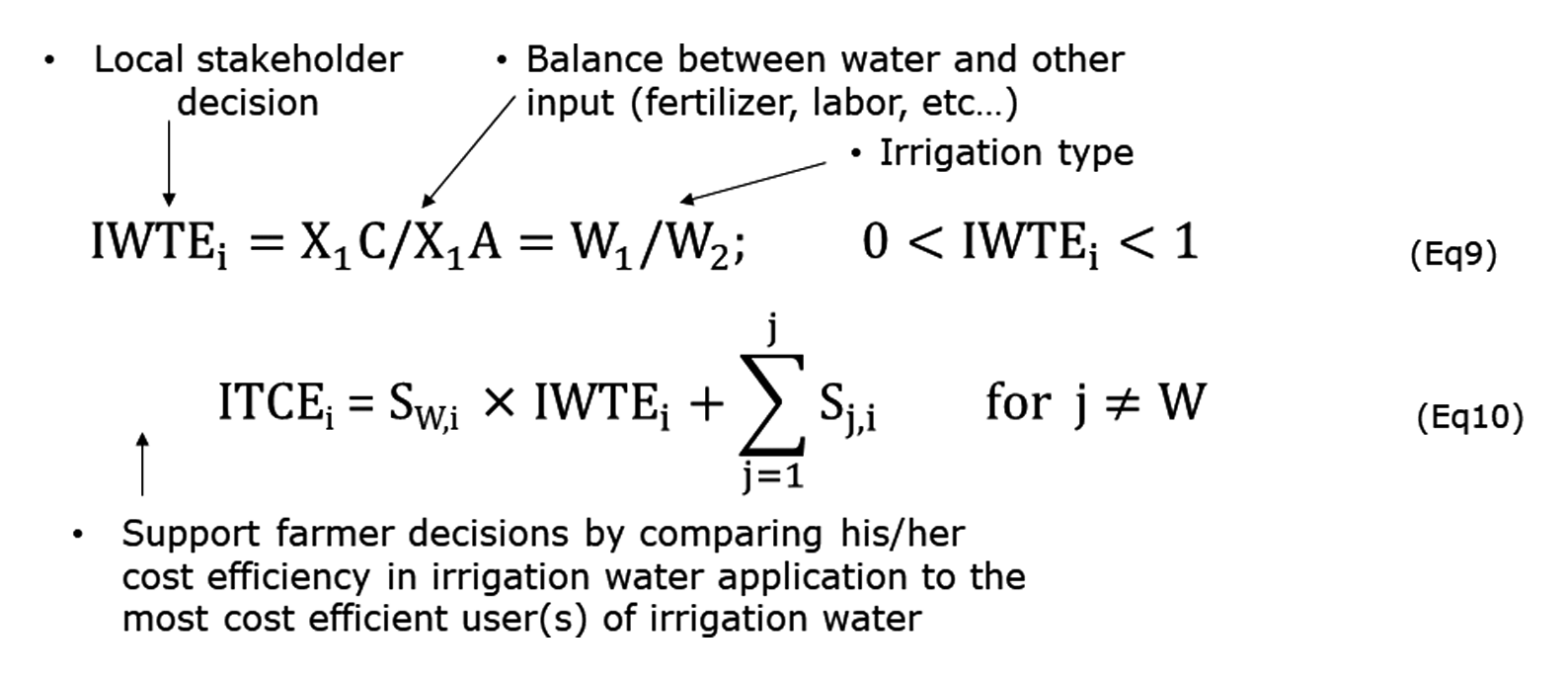

**Table:** 

	Scale/model type	Notes, references, and links
**Time-frame**	Crop cycle	
** Scale**	Farm/several fields	
** Models**	Bio-economic models and simulators such as: IMPACT; DAHBSIM	https://www.ifpri.org/publication/international-model-policy-analysis-agricultural-commodities-and-trade-impact-model-0 https://www.ifpri.org/publication/dynamic-agricultural-household-bio-economic-simulator-dahbsim-model-description

### Water efficiency, production, and resilience in arid areas

It is often assumed that better efficiency in the use of irrigation water should make it possible to safeguard water resources, improve crop production per hectare, reduce production costs by reducing irrigation inputs, potentially reduce nitrate leaching and, finally and more globally, improve farmer income. This type of assumption must be treated with great caution when the analysis is carried out at the farm level. Several dryland countries have indeed implemented support policies to promote sprinkler- and drip-irrigation instead of submersion, and this has led to higher productivity per hectare. However, intervening on the water component alone has only allowed a modest improvement of irrigation efficiency, because several other production factors have remained limiting (e.g. labour availability, adapted and certified seeds, fertility). In addition, access to water is often not available at the right time for the crop (e.g. during grain-filling). This leads to a reduction in the denominator in Eqn 9 (Box 9; quantity of irrigation water, W_2_) but a fairly small increase in the numerator, mostly expressed by the gross margin on the farm. Since irrigation rates are calculated for optimal yields and not yields limited by other factors, then a possible side-effect of not calculating the irrigation requirement correctly can be an increase of leaching as excess water is applied (El Ansari *et al.*, 2020).

Based on this, it is essential to combine the irrigation efficiency indicator with indicators that express all the ecosystem services (see also ‘WUE at the landscape level’ below), with the aim of maximizing irrigation and production while minimizing externalities. This brings us back to a trade-off analysis in which neither efficiency nor production in its different components should be considered separately. This is all the more important as the most efficient farms in the drylands today are those that are poor, with very limited access to resources (low numerator and denominator in Eqn 9.

### The limits of water efficiency

By massively subsidising the renewal of irrigation systems, dryland countries have sought to improve irrigation efficiency, increase farmer incomes, and preserve water resources; however, two unexpected effects have occurred. First, saving water per hectare has also led to an increase in the area of land that is irrigated, and hence in total water use, and second, the improvement of irrigation efficiency has either led to a simplification of the cropping system, or the partial/total replacement of traditional cropping systems based on cereals and legumes with more profitable crops. While this has led to significant increases in farmer income per hectare, it has also decreased the diversity of cropping systems on farms, and increased crop protection treatments. The quest for greater irrigation efficiency via the simplification of cropping systems has also been followed by a reduction in the diversity of food intake in farm households, leading to unbalanced diets as most or all of the production is now marketed (Chenoune *et al.*, 2017). It may seem paradoxical, but the quest for greater irrigation efficiency has been followed by a direct or indirect risk of non-resilience for farms in drylands (Souissi *et al.*, 2018; Hossard *et al.*, 2021), because these farms have either become too dependent on water (Nasrallah *et al.*, 2020), or the simplification of cropping systems that has followed the search for more efficient systems has made these systems less flexible (e.g. in the choice of crop succession or substitution) in the event of a climate shock.

## WUE at the landscape level

Going beyond the farm level is the landscape level, where it is essential to modulate water storage on the basis of trade-offs between the various and possibly antagonist water-user needs (see Box 6). Beyond satisfying those needs, modulating water levels within different compartments (e.g. root zone, aquifers, surface reservoirs) is critical for the sustainability of systems that depend on rainfall directly (infiltration for rain-fed crops) or indirectly (irrigation from aquifers or surface reservoirs).

**Box 6. WUE at the landscape scale**

WUE at the landscape scale (WUE_land_) is presented in Eqn 11 as ratios of different possible metrics, which can include gross or net primary productivity (GPP or NPP). WUE_land_ can also be expressed as an interaction function between the distribution of different crops/species within the landscape (the ‘Environment’, Envt), the management (Mgt, e.g. rainfed, irrigated, fertilized, weeded), and the crops/species chosen by farmers, averaged across the many crops/species of the landscape, and divided by ‘Water’. The latter includes several hydric indicators such as rainfall, watershed wetting (rainfall minus run-off, including infiltration for crops and underlying aquifers), root-zone water content, and withdrawals from reservoirs, and it is akin to the denominator of Eqn 5 (Box 3). The major difference with Boxes 1–4 is that WUE_land_ needs to be aggregated both in time (‘*i*’) as seen in Boxes 1–4, and in space (‘j’), and this for the many crops/species of the landscape, each occupying a *j*th portion of the space. This is developed in Eqn 12. The first part of the equation takes into account the ‘Environment’ and the ‘Management’ effects, weighted by the area covered by each combination (where *m* is the total number of combinations). The second part of the equation is akin to WUE_field_ (Box 3) and is an aggregation over time ‘i’, within each of the *j*th portions of the space occupied by a crops/species. The text labels indicate possible levers directly affecting the terms of the equations. In addition, the bold arrows and text also indicates unavoidable interactions/trade-offs; for instance with the WUE of other adjacent landscapes, between the landscape design and non-farming stakeholders, and with the need for ecosystem services such as river flow.

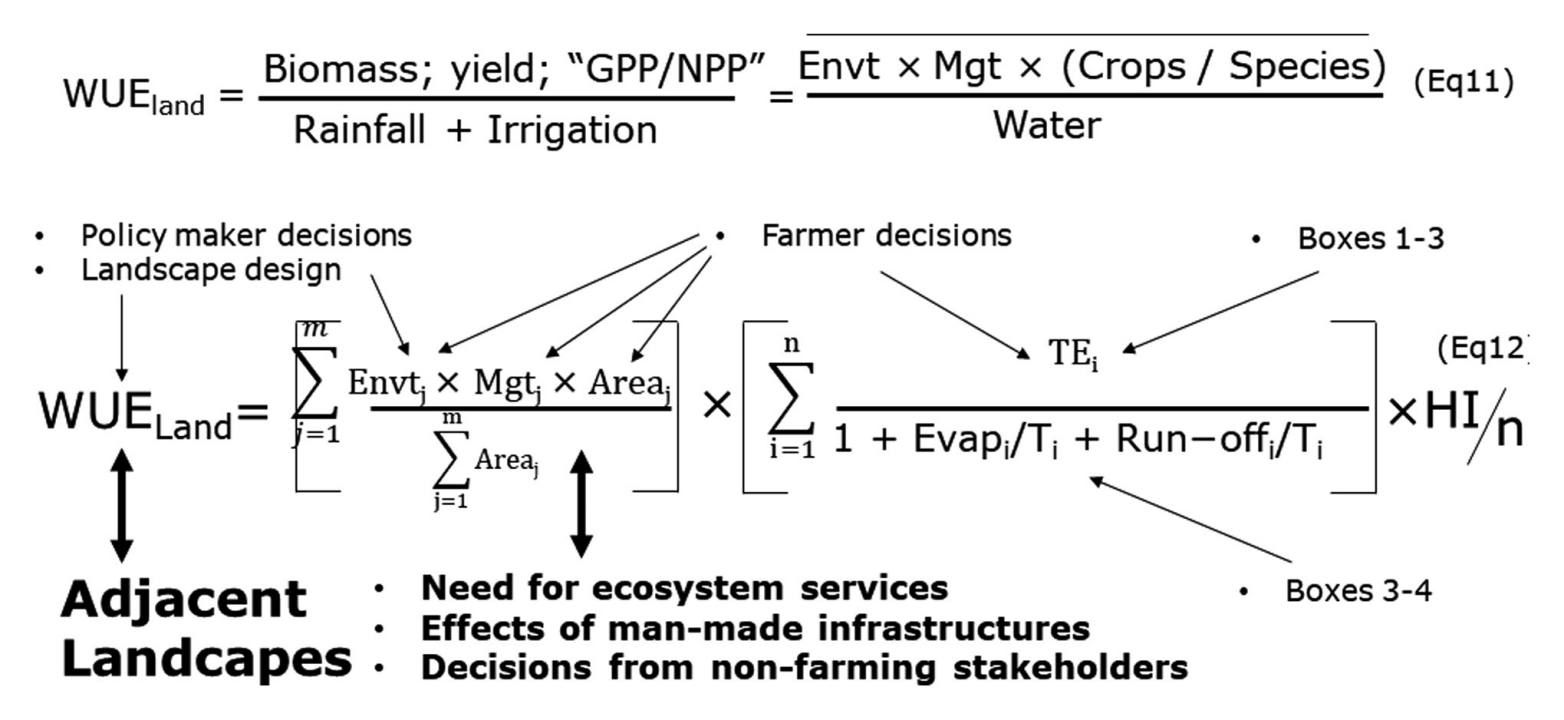

**Table:** 

	Scale/model type	References and links
**Time-frame**	Day(s) to phenological stages, from one to several crop cycles, up to decades	
**Scale**	Fields, land-use classes, watersheds, aquifers	
**Models**	Hydrological and agro-hydrological, models such as: SWAT; ParFlow; APEX or Coupled hydrological and crop growth models Integrated models such as MHYDAS-Small-Reservoirs Watershed and land-management models such as WEAP	https://swat.tamu.edu/ https://parflow.org/ https://epicapex.tamu.edu/ Lebon *et al.* (2022) https://www.sei.org/projects-and-tools/tools/weap/

### Landscape level: definition, concepts, and levers of action for WUE

The notion of ‘landscape’ carries different meanings and its definitions are numerous (Aznar *et al.*, 2006). Landscape is considered in this review as a portion of a territory from a few km² to a few tens of km². Agricultural landscapes are characterized, among other things, by their composition and configuration (Liu *et al.*, 2020). The composition includes the different types of land uses and crops over agricultural soils, as well as various man-made infrastructures that influence water flows. The configuration is the way in which the composition is organized in space. Composition and configuration of agricultural landscapes evolve over time, according to farmer choices. The composition then represents an important lever for WUE at the landscape scale (WUE_land_), as this is where the choice can be made between water-efficient and -inefficient crops (Box 6).

Analysing and controlling WUE_land_ requires understanding various water uses and related ecosystem services: production of blue water for agricultural, domestic, and industrial uses, availability of green water for non-cultivated terrestrial ecosystems (e.g. forest, scrubland), and preservation of aquatic environments (e.g. lakes, rivers, wetlands). In doing so, the analysis of water availability and uses leads to the delineation of landscapes according to the watershed, a hydrological spatial unit that enables the determination of the drivers of soil water availability such as hydrological fluxes (infiltration, run-off, evaporation, transpiration, groundwater recharge) and their interactions within the hydrological cycle (Vereecken *et al.*, 2015). Increasing the WUE of crops might have a negative impact on other water uses within the watershed that therefore have to be both taken into account and quantified. In other words, at the landscape level, the challenge is to establish trade-offs between one or more ecosystem functions of the soil water supply that we seek to modulate in time and space to increase the WUE of crops, and between other ecosystem functions that we also seek to modulate, such as variations the flow of streams. The landscape approach to WUE must therefore be part of a global approach of water management (Habibi Davijani *et al.*, 2016; Psomas *et al.*, 2016), within which water is considered as a resource for agriculture and other human uses, but also as a living environment for plants and animals. The approach therefore requires involving a diversity of stakeholders and social groups with diverse and possibly antagonistic interests (e.g. national and local authorities, water production and distribution companies, fishermen, environmental non-governmental organizations), a diversity that goes far beyond the circle of farmers or groups of farmers (Koontz *et al.*, 2014).

The landscape-specific levers for steering and optimizing WUE_land_ are two-fold (Box 6). The first lever is the choice of landscape composition (Stroosnijder *et al.*, 2012). This involves first determining the crop species and varieties adapted to the climatic conditions and soil-water availability, second specifying agricultural practices to be implemented for modulating rainwater infiltration or limiting evaporation (e.g. type of ploughing and date, seeding density and date, grassing, mulching), and third determining effective man-made infrastructures to appropriately distribute rainwater between landscape compartments (Eqn 12, Box 6). The main landscape infrastructures are rainwater harvesting systems such as reservoirs, terraces, and contour trenches or ridges that follow the topographic levels (Habets *et al.*, 2018; Lasage *et al.*, 2015). The second lever for controlling WUE_land_ consists of determining the landscape configuration, namely the spatial allocation of crops and practices at the scales of sub-fields and field patchworks (Colin *et al.*, 2012), and the implementation of landscape infrastructures in specific areas related to the pedological substrate and hydrographic network. This determination relies on scientific or expert knowledge about the environment, climate, and hydrology across the whole landscape (Laudon *et al.*, 2018). Beyond water resource management, determining the landscape composition and configuration is the basis of landscape agroecology (Jeanneret *et al.*, 2021) (Eqn 12, Box 6).

### WUE at the landscape level: biophysical metrics and scales

WUE_land_ can be quantified in relation to the water pathways at the scale of a local watershed of a few km^2^ to a regional watershed of a few tens of km^2^ (Wilson *et al.*, 2022) (Box 6). Because WUE_land_ aggregates WUE from the many crops/vegetation within the landscape, the aggregation is at scales of both time (Boxes 1–) and space. Aggregation at small scales of space include the ratios presented in Boxes 3 and 4, and these are expanded to gross/net primary productivity to plant transpiration or crop evapotranspiration at the scale of an agricultural field, and over time-scales that range from days to the full crop cycle (Box 6). Aggregation at larger spatial scales will include a mix of rain-fed and irrigated crops at different sizes and rely on a variety of hydric indicators such as rainfall, watershed wetting (the difference between rainfall and run-off, and including watershed-scale infiltration for crops and underlying aquifers), root-zone water content for crops only, and withdrawals from reservoirs used for irrigation (Du *et al.*, 2018; Abeshu *et al.*, 2021). These large-scale aggregations can be placed into different categories and allow the measurement of efficiency at different scales (land-use classes, watersheds, aquifers). These large-scale metrics are also defined across various time-scales, from days to the whole crop-cycle, including specific periods related to phenological stages when the balance between water needs and water availability is critical, as discussed above (e.g. grain-setting at the beginning of spring for rain-fed crops under semi-arid climates) (see Box 3).

### Current research paths

WUE can be evaluated using actual data obtained by monitoring the implementation and subsequent impacts of new cropping systems or landscape infrastructures (*ex post* approaches), or using forecasts to evaluate the potential impacts of changes in landscape composition/configuration (*ex ante* approaches). *Ex post* evaluation allows changes to be assessed experimentally in real conditions with non-academic stakeholders, whilst *ex ante* approaches can provide guidance to stakeholders in the context of long-term adaptations linked to global changes. Both approaches have to be considered simultaneously in the design and assessment of integrated water management policies (Hashemi *et al*, 2019).

At the landscape scale, the current challenge is to evaluate WUE_land_ both from an integrated viewpoint across the watershed, which is the analysis/decision level of interest for decision-makers, and from a local viewpoint from the perspective of the agricultural field, which is the analysis/decision level for farmers. This scientific challenge presents various research avenues (Eqn 12, Box 6). The first avenue is the development of observation methods at defined spatial scales for characterizing the landscape composition and configuration, including classes, delineations, geometries, and functional properties, and for characterizing these variables in relation to WUE indicators, namely rainfall, evapotranspiration, run-off, and soil moisture. Recent research has focused on various innovations and limitations related to the assessment of these variables using remote sensing (Jacob *et al.*, 2014; Weiss *et al*, 2020; Deliry *et al.*, 2021; Chen *et al.*, 2022). The second avenue is the design of agro-hydrological models that simulate both crop functioning and water fluxes within a landscape in a coupled manner (e.g., Lebon *et al.*, 2022). Agro-hydrological models are potential tools for *exante* assessment of WUE at the landscape scale when evaluating possible choices of cropping systems or landscape management modes in accordance with predicted climate scenarios (Krysanova *et al.*, 2015). The third avenue is the formulation of scenarios about how the landscape composition and configuration will evolve. Such scenarios, which should be compatible with the formalisms of agro-hydrological models for evaluation purposes, require the design of landscape modeling tools that can be used with participative protocols in order to take into account the drivers of stakeholder strategies (De Girolamo and Lo Porto, 2012).

## Conclusions

It is a long way from intrinsic transpiration efficiency at the leaf level to the improvement of water use efficiency at farm system/landscape level. In this review we have attempted to draw a path and show the connections, as well as the trade-offs, between increasing biological, physical, hydrological, and human scales. While additive efficiency gains can be made at each of these different scales, overall gains in water use efficiency can only be made if the scales are connected and if the numerous trade-offs along the way are meaningfully addressed. In this context, human decisions, often moulded by societal/policy influences, very likely represent the main level of influence on landscape WUE, for example the choice of a water-efficient crop species, a water-efficient irrigation system, or a landscape allocation/design that will maximize return on water. However, even when these choices are made, there remains a lot of room for WUE improvement from the plant/crop perspective, following the agronomic levers presented in Box 3, and using water-efficient cultivars developed from plant traits described in Boxes 1 and 2. Equation 12 in Box 6 is our attempt to put together the levers that exist to increase WUE from the organ/plant/crop/field perspective (Boxes 1–4) with the aspects of the human and societal dimension that only appear when plants/crops become part of a landscape, and to highlight necessary trade-offs (for instance for ecosystem services) within and beyond the landscape scale.

## Supplementary data

The following supplementary data are available at *JXB* online.

Appendix S1. The availability and quality of data as an important limiting factor in the analysis of water use efficiency.

erad052_suppl_Supplementary_Appendix_S1

## Data Availability

No new data were generated in this review.
