# Supplementary material for: Water use efficiency across scales: from genes to landscapes
Source: J Exp Bot. 2023 Feb 13;74(16):4770–88. doi: 10.1093/jxb/erad052 (PMC10474597; doi:10.1093/jxb/erad052)
Supplement: erad052_suppl_Supplementary_Appendix_S1 [file erad052_suppl_supplementary_appendix_s1.pdf]

Appendix S1.

Data, an important limiting factor in the efficiency analysis when using multi-criteria and multi-scale approaches - The databases used for the calculation of water efficiency are related to the purpose, domain and spatial scale of the study. Two scales/domains are often cited in the literature but often separately: i) the field as a base element where soil-plant processes (water and nutrient budgets, yield formation...), influenced by climate and management, occur, and ii) the farm as the level where field decisions are made, where production is related to labour availability, the market, natural resource use and family food consumption, and where policies influence decisions (subsidies, penalties, etc.). In most of the above studies, the proposed methods do not take into account the possible interactions between the biophysical characteristics of the farm and the socio-economic resources and strategies that determine the productive potential and efficiency of the system at field and farm levels (Belhouchette *et al.*, 2012). In fact, every decision made at a particular level of this complex hierarchical system (field, farm) potentially affects the functioning of the other levels negatively and/or positively (Souissi *et al.*, 2018) and the efficiency of the whole system may arise following a specific combination of diversity at the different levels (plant diversity in the fields and activity diversity on a farm) (El Ansari *et al.*, 2020).
For a multi-domain and multi-scale efficiency analysis, Hammouda *et al.*, 2018 suggested building the efficiency related-database by combining a list of interconnected socio-economic (labour availability, crop contracts, etc.), agronomic, as well as technical (rotation, irrigation, soil, etc.) components/variables, using a system approach. To do so, the concept of Agricultural Activity, which is based on an approach known as "primal", is suggested (Flichman *et al.*, 2011; Hammouda *et al.*, 2018). This approach makes it possible to establish a direct relationship between the choice of inputs (in terms of quantity or timing) and their effects on production and externalities/services, for every cropping system used. This approach differs from the conventional economic approach within which the Agricultural Activity is represented by a production or a cost, without taking into account the way in which this activity is carried out, and without specifying the biophysical context (soil, climate) in which it takes place (Louhichi *et al.*, 2010). This conventional approach therefore implies that all the Agricultural Activities which have the same production level and/or production costs are not only substitutable, but they present the same crop management practices, as well as the same externalities, potentially (Harper and Zilberman, 1989). Similarly, this implies that the production and externalities produced are directly correlated with the cost of production, and not with their production process (Flichman and Jacquet, 2003).
In order to define each Agricultural activity, it is important to have quantifiable data on the inputs used for each activity, as well as on its outputs (production and externalities). These data can come from different sources: experimentation, experts, farmer surveys, regional statistics. However, this often seems insufficient for the multi-criteria and multi-scale analysis of efficiency. For this reason, Belhouchette *et al.*, 2011 suggest the use of biophysical modelling (in particular by using summary models) and bio-economic modelling to generate data in order to better understand the link between

biophysical data and technical and economic data.

References

Belhouchette H, Blanco M, Wery J, Flichman G. 2012. Sustainability of irrigated farming systems in a Tunisian region: A recursive stochastic programming analysis. Computers and Electronics in Agriculture **86,** 100-110.

Belhouchette H, Louhichi K, Therond O, Mouratiadou I, Wery J, van Ittersum M, Flichman G. 2011. Assessing the impact of the nitrate directive on farming systems using a bio-economic modelling chain. Agriculture Systems **104** (2), 135-145

El Ansari L, Chenoune R, Yigezu YA, Gary C, Belhouchette H. 2020. Trade-offs between sustainability indicators in response to the production choices of different farm household types in drylands. Agronomy, MDPI, **10** (7), pp.998.

Flichman G. 2011. (Ed.), Bio-Economic Models applied to Agricultural Systems, Springer Netherlands, Dordrecht (2011), pp. 3-14, 10.1007/978-94-007-1902-6_1

Flichman G, Jacquet F. 2003. Le couplage des modèles agronomiques et économiques : intérêt pour l'analyse des politiques. Cahiers d'Economie et de Sociologie Rurales **67**, 51-69

Hammouda M, Wery J, Darbin T, Belhouchette H. 2018. Agricultural activity concept for simulating strategic agricultural production decisions: case study of weed resistance to herbicide treatments in South-West France. Computers and Electronics in Agriculture **155**, 167-179

Harper CR, Zilberman D. 1989. Pest externalities from agricultural inputs. American Journal of Agriculture Economics **71** (3), 692-702

Souissi I, Boisson J, Mekki I, Therond O, Flichman G, Belhouchette H 2018. Impact assessment of climate change on farming systems in the South Mediterranean area: a Tunisian case study. Regional Environmental Change, Springer Verlag, **18** (3), 637-650
